# Limited Changes in Lifestyle Behaviours after Non-Muscle Invasive Bladder Cancer Diagnosis

**DOI:** 10.3390/cancers14040960

**Published:** 2022-02-15

**Authors:** Ivy Beeren, Liesbeth de Goeij, Rana Dandis, Nikoletta Vidra, Moniek van Zutphen, J. Alfred Witjes, Ellen Kampman, Lambertus A. L. M. Kiemeney, Alina Vrieling

**Affiliations:** 1 Department for Health Evidence, Radboud University Medical Center, 6500 HB Nijmegen, The Netherlands; ivy.beeren@radboudumc.nl (I.B.); liesbethdegoeij@hotmail.com (L.d.G.); rana.dandis@radboudumc.nl (R.D.); nikolettavidra@gmail.com (N.V.); moniek.vanzutphen@radboudumc.nl (M.v.Z.); bart.kiemeney@radboudumc.nl (L.A.L.M.K.); 2 Department of Urology, Radboud University Medical Center, 6500 HB Nijmegen, The Netherlands; fred.witjes@radboudumc.nl; 3 Division of Human Nutrition and Health, Wageningen University & Research, 6708 WE Wageningen, The Netherlands; ellen.kampman@wur.nl

**Keywords:** non-muscle invasive bladder cancer, lifestyle changes, lifestyle recommendations, trajectory analysis, cancer survivorship

## Abstract

**Simple Summary:**

Little is known about lifestyle behaviours following a diagnosis with non-muscle invasive bladder cancer. In this observational study, we aimed to investigate adherence to lifestyle recommendations and changes in lifestyle in 935 patients with non-muscle invasive bladder cancer in the first fifteen months after diagnosis. Second, we aimed to identify distinct trajectories of lifestyle change and their correlates. Each patient was assigned a score based on adherence to lifestyle recommendations focusing on diet, body mass index, and physical activity. We found that patients’ adherence to these lifestyle recommendations was low. Their overall lifestyle score remained constant up to fifteen months after diagnosis. Small to moderate changes in and different trajectories of single lifestyle behaviours were observed, with correlates differing per trajectory group. Given that there is considerable room for lifestyle improvement, effective strategies to support favourable lifestyle changes are needed.

**Abstract:**

The aim of our study was to investigate adherence to lifestyle recommendations and lifestyle changes after diagnosis in patients with non-muscle invasive bladder cancer (NMIBC). Second, we aimed to identify distinct trajectories of lifestyle change and their correlates. We analysed data of 935 patients with NMIBC from a prospective cohort study at six weeks (evaluating pre-diagnostic lifestyle), three months, and fifteen months after diagnosis. An overall lifestyle score (range 0–7) was calculated based on the 2018 World Cancer Research Fund/American Institute for Cancer Research (WCRF/AICR) recommendations focusing on diet, body mass index, and physical activity. Linear mixed models were used to analyse absolute lifestyle changes over time. Distinct trajectories of change were identified with latent class trajectory models. We found an overall lifestyle score of 3.3 which remained constant over time. The largest lifestyle changes were observed for the consumption of red and processed meat (−96 g/week) and fruit and vegetables (−38 g/day). Two to four trajectory groups were identified for each single lifestyle behaviour. Correlates differed per trajectory group. In conclusion, adherence to the WCRF/AICR recommendations was low. Small to moderate changes in and different trajectories of single lifestyle behaviours were observed. Effective strategies for lifestyle improvement are warranted.

## 1. Introduction

Bladder cancer is the tenth most commonly diagnosed malignancy worldwide and the sixth most common in males [1]. About 75% of all incident cases presents with non-muscle invasive bladder cancer (NMIBC, stages Ta, Tis, and T1) [2]. Since patients with NMIBC are at high risk of disease recurrence, intensive treatment and follow-up programs are required that impose a large burden on patients and healthcare resources [3,4,5]. Given the chronic nature of NMIBC, it is important to explore the role of modifiable lifestyle behaviours, such as diet, body weight, physical activity, and smoking, during the disease course [6,7].

While evidence is not yet available for patients with NMIBC, a healthy lifestyle is linked to the prognosis of several common malignancies [8]. For now, evidence is insufficient to formulate specific lifestyle recommendations for cancer survivors. Hence, cancer survivors are encouraged to follow the cancer prevention recommendations of the World Cancer Research Fund/American Institute for Cancer Research (WCRF/AICR) [9]. A pooled analysis showed that adherence to multiple lifestyle recommendations is generally low in cancer survivors, while adherence to single lifestyle behaviours is often higher [10]. While our previous analysis indicated that patients with NMIBC did not adhere to alcohol and body mass index (BMI) recommendations both before and 3 months after diagnosis, the majority did adhere to smoking and physical activity recommendations. Dietary factors were not included in these analyses yet [11], although a cross-sectional study found that bladder cancer survivors were often not meeting lifestyle recommendations concerning diet, BMI, and physical activity [12].

It has been argued that a cancer diagnosis may trigger favourable lifestyle changes shortly after diagnosis, but results differ by lifestyle behaviour [13,14,15,16,17,18,19]. Most prospective observational studies reported smoking cessation and improvements in diet after cancer diagnosis [14,15,16,17,18,19], including an increased intake of fruit and vegetables and a decreased intake of red and processed meat, sugary drinks and alcohol. Results for BMI and physical activity are less consistent, as increases, decreases and no changes were observed [13,14,15,17,18,19]. Since the analytical approach of these studies was often limited to testing for average changes between two or more timepoints, potential differences in changes between subgroups may have been overlooked [20]. Aiming to identify distinct subgroups who follow similar patterns of change (i.e., trajectories) over time would be useful to gain a better, more comprehensive, understanding of longitudinal lifestyle modification. These trajectories can then be used to identify which sociodemographic and clinical patient characteristics (i.e., correlates) are associated with favourable or unfavourable lifestyle trajectories to recognize patients who can benefit most from lifestyle support.

The first aim of our study was to assess adherence to the WCRF/AICR recommendations and changes in overall lifestyle and single lifestyle behaviours in the first fifteen months after NMIBC diagnosis. Second, we aimed to identify distinct trajectories of change for overall lifestyle and single lifestyle behaviours, as well as correlates of these trajectories.

## 2. Methods

### 2.1. Study Population

This study is part of the UroLife study (Urothelial cell cancer: Lifestyle, prognosis and quality of Life), a prospective cohort study in patients with newly diagnosed NMIBC. Patients were recruited between May 2014 and April 2017 in one of 22 participating hospitals in the Netherlands [21]. Eligible patients were identified through the Netherlands Cancer Registry using notification lists of the Pathological Anatomical National Automated Archive (PALGA Foundation). Patients were eligible if they were between 18 and 80 years old, Dutch speaking, diagnosed with a histologically confirmed primary stage Ta, Tis, or T1 NMIBC tumour, and underwent a transurethral resection (TUR). Patients who were diagnosed with cancer in the past five years, those who had lymph node or distant metastases, and those not at risk of tumour recurrence (follow-up < 90 days) were excluded from participation. All participants provided written informed consent. Ethical approval was provided by the Committee for Human Research region Arnhem-Nijmegen (CMO 2013-494) on 17 January 2014.

The current study used data of participants with two or three complete assessments of each lifestyle behaviour, resulting in four slightly different analytic populations to study overall lifestyle, diet, BMI, physical activity, and smoking.

### 2.2. Data Collection

Information on diet, body weight, physical activity, and smoking status was collected via self-reported web-based or paper-and-pencil-based questionnaires at six weeks (T6wk), three months (T3mo), and fifteen months (T15mo) after diagnosis. Data that were collected at T6wk reflected lifestyle before diagnosis (baseline).

Habitual dietary intake was assessed using a 163-item validated food frequency questionnaire (FFQ) developed by Wageningen University [22,23,24]. The reference period was the previous year (T6wk) or the previous month (T3mo and T15mo). The absolute intake of every item was calculated by combining frequencies of intake with standard portion sizes and household measures. The 2011 Dutch Food Composition Database [25] was to calculate dietary fibre, alcohol and total energy intake.

Other lifestyle questionnaires included questions regarding body weight, physical activity, and smoking status. BMI was calculated based on body weight (at T6wk, T3mo, and T15mo) and height (at T6wk only) and computed in kg/m^2^. Habitual physical activity was assessed through the validated short questionnaire to assess health-enhancing physical activity (SQUASH) [26,27,28], with the reference period being a normal week in the months before diagnosis (T6wk) or the previous month (T3mo and T15mo). Moderate-to-vigorous physical activity included leisure time (cycling, gardening, odd jobs, and sports) and commuting (walking and cycling) activities with a metabolic equivalent value (MET) of ≥3.0 [29], but not household and work activities.

In addition, data on demographics and clinical factors were collected. Information on demographics was collected through a questionnaire at T6wk and included information on age, gender, highest education level, living situation, and occupation status. The presence of 14 comorbidities was evaluated with an adapted version of the Self-administered Comorbidity Questionnaire (SCQ) [30]. Information on tumour characteristics was retrieved from medical records by data managers of the Netherlands Cancer Registry. For these analyses, we used incidence date, tumour stage, differentiation grade, and recurrence/progression status (up until T15mo).

### 2.3. WCRF/AICR Recommendations Adherence Score

Adherence to the 2018 WCRF/AICR recommendations of cancer prevention was quantified using the standardized score as developed by Shams-White et al. [31,32] and was used as an indicator for overall lifestyle. We operationalized seven recommendations (Table 1), focussing on BMI, physical activity, fruit and vegetables, dietary fibre, ultra-processed foods, red and processed meat, sugary drinks, and alcohol. For each component, we assigned a score of 1 (full adherence), 0.5 (partial adherence), or 0 (low adherence). One recommendation (eating a diet rich in wholegrains, vegetables, fruits, and beans) was separated into two sub-recommendations. Given that the total score per recommendation could not exceed 1, each sub-recommendation was awarded with a weighted score of 0.5 (full adherence), 0.25 (partial adherence), or 0 (low adherence). Pre-defined cut-off criteria were used for all recommendations, with the exception of ultra-processed foods. Classification of ultra-processed foods was based on an adapted version of the NOVA classification on food processing [31,32]. Cut-offs for this recommendation were determined by cohort-specific tertiles calculated from the percentage of total energy intake that was derived from ultra-processed foods at T6wk [33]. The final score ranged from 0 to 7, with higher scores indicating better adherence to the WCRF/AICR recommendations. The WCRF/AICR adherence score was calculated at T6wk, T3mo, and T15mo.

### 2.4. Statistical Analyses

Linear mixed models were applied to examine changes in lifestyle over time. The WCRF/AICR score as well as all single lifestyle behaviours (consumption of fruit and vegetables, dietary fibre, ultra-processed foods, red and processed meat, sugary drinks, alcohol, physical activity, and BMI) were modelled separately using two or three repeated measurements at T6wk, T3mo, and T15mo. Time since diagnosis (in months) was included in each model as a categorical variable. A random intercept was used in all models. Except for physical activity, all models also included a random slope, since it significantly improved the model, as assessed by a log likelihood ratio test. The estimated marginal means and effect sizes for the WCRF/AICR score and each single lifestyle recommendation were derived from the linear mixed models. For smoking, a logistic mixed model was used.

Subsequently, we used latent class trajectory models to identify trajectories of change, using the framework of Lennon et al. [34]. Similar to the linear mixed models, we modelled the overall WCRF/AICR score as well as all single lifestyle behaviours. The most optimal models were selected through stepwise comparison of the Bayesian Information Criterion (BIC) of models consisting of one to seven trajectory groups [34,35]. The models with the lowest BIC and with all groups including ≥5% of the total study population were favoured. Then, the adequacy of the selected models was evaluated by the average posterior probability of assignment (≥70%) and odds of correct classification (≥0.5) of every trajectory group [34]. To ensure groups were distinguishable, each trajectory plot was visually checked for reasonably narrow and non-overlapping confidence intervals (CIs). When data were poorly fit by the normal distribution, we applied a beta cumulative distribution function in order to transform the data. To avoid making any assumptions about the covariance structure, we used a non-structured covariance matrix that was allowed to differ across groups. Trajectory groups were labelled by the relative baseline level (zero, low, medium, high) and the direction of change (increaser, decreaser, maintainer).

Multinomial logistic regression analyses were used to identify correlates of lifestyle trajectory membership. All models included age at diagnosis (18–64 years or ≥65 years), gender (male, female), educational level (low, medium, high), living situation (with partner, with partner and kids, alone with or without kids), smoking status (never, former, current), tumour stage (Ta or Tis/T1), and the presence of comorbidities (0, 1, ≥2). BMI (normal weight (<25.0 kg/m^2^), overweight (25.0–29.9 kg/m^2^), obese (≥30.0 kg/m^2^)) was also included in the models, except for the BMI trajectories. All correlates were included in the models simultaneously to determine the contribution of each correlate, while controlling for the others. For multinomial logistic regression, the “least successful” trajectory group of each lifestyle behaviour was selected as the reference category. The trajectory that deviated most from the WCRF/AICR recommendations was labelled as “least successful”. Odds ratios (ORs) were calculated together with the corresponding CIs.

To evaluate the robustness of our results, a sensitivity analysis was performed by restriction to participants with complete data at all three time points. In another sensitivity analysis, we excluded observations with extreme energy intakes (for women <500 or >3500 kcal/day, for men <800 or >4200 kcal/day) and extreme intakes of fruit and vegetables, dietary fibre, ultra-processed foods, red and processed meat, sugary drinks, or alcohol (≥3 standard deviations from the mean). To evaluate whether changes in lifestyle over time were different for patients who did or did not experience disease recurrence or progression before filling out the questionnaire at T15mo, we added an interaction term between recurrence and progression status (yes or no) and time in the mixed models of the WCRF/AICR score and all single lifestyle behaviours.

All analyses were performed in R version 3.6.2 [36]. The lme4, lcmm, and nnet packages were used for linear mixed models, latent class trajectory models, and multinomial logistic regression analyses, respectively. Two-sided *p* values of <0.05 were considered statistically significant.

## 3. Results

### 3.1. Study Population

**Figure 1:**
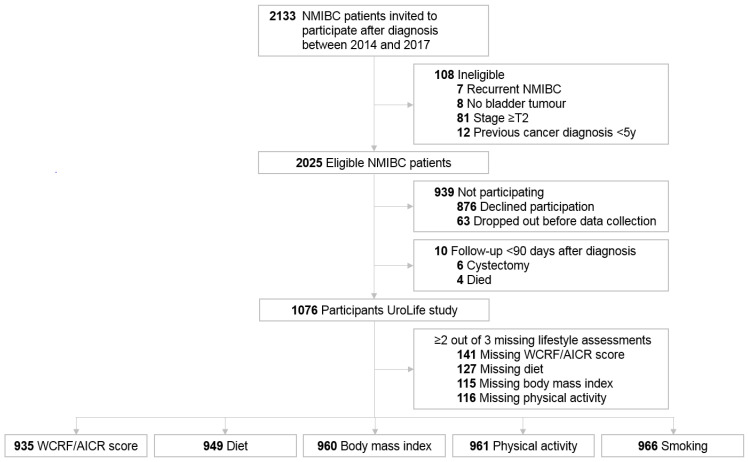
Flowchart of the study population in a longitudinal study among non-muscle invasive bladder cancer patients (NMIBC), the UroLife study.

Of the 2133 invited patients, 2025 patients were eligible and 1076 (53%) agreed to participate in the UroLife study (Figure 1). A total of 935 participants were included in the analyses of the WCRF/AICR score, while the analytic populations for diet (*n* = 949), BMI (*n* = 960), physical activity (*n* = 961), and smoking (*n* = 966) were slightly larger.

Baseline characteristics of the included and excluded participants in the WCRF/AICR score analyses are shown in Table 2. Included participants were predominantly male (80%), had a low education level (48%) and were retired (61%). The median (IQR) overall WCRF/AICR score was 3.3 (2.8–3.8) at baseline. Nearly half of the participants were overweight (47%), and most of them were former smokers (59%). Most participants had two or more comorbidities (59%) and stage Ta disease (76%). In comparison, excluded participants were more often low educated (57%) and had a lower median WCRF/AICR score of 2.8 (IQR 2.5–3.5).

### 3.2. Adherence to WCRFF/AICR Recommendations

**Figure 2:**
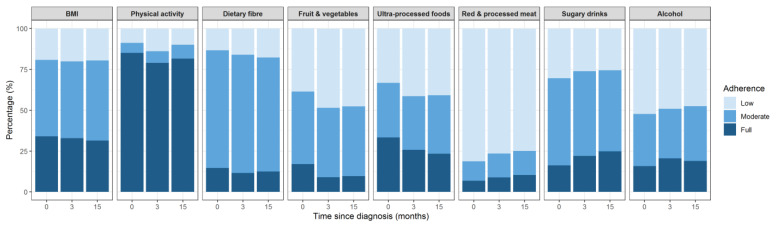
Adherence to the 2018 World Cancer Research Fund/American Institute for Cancer Research (WCRF/AICR) lifestyle recommendations at baseline, three months and fifteen months after diagnosis in 935 patients with non-muscle invasive bladder cancer.

Overall, large variation was observed in adherence between the different lifestyle recommendations (Figure 2). Adherence to the dietary recommendations was generally low. Apart from the recommendation for ultra-processed foods (23–33%), less than 25% of the participants reported full adherence with the dietary recommendations at any time point. The recommendation to limit red and processed meat intake showed the lowest adherence (7–10%). The majority (79–85%) fully adhered to the recommendation to be physically active. About one-third of the participants had a BMI within the healthy range.

### 3.3. Lifestyle Changes

While statistically significant changes were observed in all single lifestyle recommendations except for BMI, the mean WCRF/AICR score remained constant at 3.3 (Table 3). The largest improvement was observed for red and processed meat intake (−96 g/week).

Additionally, consumption of ultra-processed foods (−2.2 en%), sugary drinks (−28 g/day), and alcohol (−2.3 g/day) and the proportion of current smokers (−8.1%) changed in concordance with the WCRF/AICR recommendations. In contrast, the decline in fruit and vegetables intake (−38 g/day), dietary fibre intake (−1.4 g/day), and physical activity (−85 min/week) was not in line with the recommendations. Small to moderate effect sizes were observed that ranged between −0.49 and 0.38.

### 3.4. Trajectories of Change and Their Correlates

**Figure 3:**
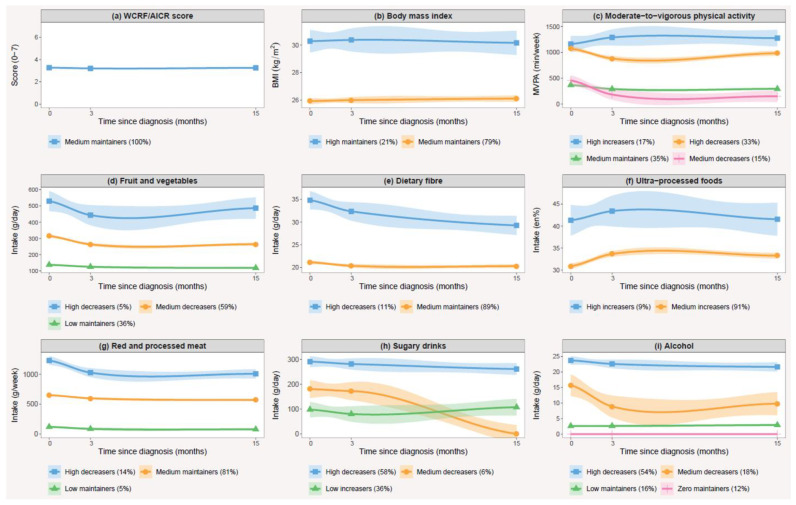
Mean and 95% confidence intervals of lifestyle behaviours by trajectory groups during the first fifteen months after diagnosis with non-muscle invasive bladder cancer. This includes trajectory groups of (**a**) the WCRF/AICR score; (**b**) body mass index; (**c**) moderate-to-vigorous physical activity; and the intakes of (**d**) fruit and vegetables; (**e**) dietary fibre; (**f**) ultra-processed foods; (**g**) red and processed meat; (**h**) sugary drinks; (**i**) alcohol.

Two to four trajectory groups were identified for each of the eight single lifestyle behaviours, while no distinct trajectories were observed for the overall WCRF/AICR score (Figure 3). The trajectories were mainly characterized by their relative baseline level, but differences in the shape of the trajectory curves were also observed, consisting of decreasers, maintainers and increasers of specific lifestyle behaviours. The model-specific and class-specific statistics used for assessing relative adequacy are summarized in Tables S1 and S2, respectively. All models met the most important criteria for model fit.

Apart from BMI, trajectories of all other lifestyle behaviours showed associations with one or more patient characteristics (Tables S3–S10). The correlates were different for each trajectory group as well as for each lifestyle behaviour. Gender and level of education were often associated with trajectory group membership. In most cases, females were more likely to belong to more favourable trajectory groups, except for trajectories of dietary fibre and physical activity, where a reverse association was observed. A higher education level was generally associated with membership to more favourable trajectory groups, except for alcohol intake, where those with a higher education level were less likely to belong to a more favourable trajectory group.

### 3.5. Sensitivity Analyses

No substantial different results were observed when restricting to participants with complete data at all three time points (*n* = 737 for WCRF/AICR score, *n* = 795 for diet, *n* = 814 for BMI, *n* = 784 for physical activity, *n* = 817 for smoking) in the outcomes of the linear mixed models (Table S11). The results of the latent class trajectory models also remained similar, yet we identified one trajectory group less for both physical activity and red and processed meat (data not shown). Results of the linear mixed models also did not differ when excluding observations with extreme energy or dietary intakes (*n* = 68 for WCRF/AICR score and *n* = 58 for diet) (Table S12). No considerable different results were observed in the latent class trajectory models, although we identified one trajectory group less for both red and processed meat and fruit and vegetables intake (data not shown). In all cases, the interpretation of the trajectory curves remained similar. No statistically significant differences in changes in the WCRF/AICR score and individual lifestyle behaviours were observed between participants who did (*n* = 137) and did not experience recurrence or progression (all P_interaction_ > 0.05).

## 4. Discussion

In this prospective cohort study, we found low adherence to the WCRF/AICR lifestyle recommendations among patients with NMIBC. Although no changes were observed in the overall WCRF/AICR score in the first fifteen months after diagnosis, changes were found for single lifestyle behaviours. The largest differences were noted for the consumption of red and processed meat, fruit and vegetables and current smoking status, all showing a declining trend over time. Two to four trajectory groups were identified for all eight lifestyle behaviours, but not for the overall WCRF/AICR score. The trajectory groups mostly differed by baseline level, yet differences in the shape of trajectory curves were also observed. Correlates differed per trajectory group, but often included gender and education level.

No previous study has investigated adherence to the WCRF/AICR recommendations in NMIBC patients, but studies in other cancer survivors also reported low to moderate adherence to these recommendations [10,14,15,37]. A pooled analysis provided estimates of adherence to single 2007 WCRF/AICR recommendations after a cancer diagnosis in studies that mainly included breast and colorectal cancer patients [10]. Although adherence to the BMI recommendation (39%) was in line with our findings, higher adherence rates for dietary recommendations were observed in the pooled analysis (31% to 47%), and lower adherence to the physical activity recommendation was reported (43%) [10]. Differences in questionnaires, study population and in definitions of adherence could explain some of the inconsistencies between studies. When we compare our results with studies in Dutch cancer survivors, the results are more similar. In these studies, adherence to dietary recommendations was generally low (<35%), and the majority adhered to the physical activity recommendation (50–90%) [15,38].

This was the first study to examine lifestyle changes in patients with NMIBC. Two other prospective cohort studies reported small but significant changes in the WCRF/AICR score after cancer diagnosis [14,15]. One study in 1072 Dutch colorectal cancer patients observed a marginal, although statistically significant, improvement in the WCRF/AICR score of 3.4 at baseline to 3.5 in the first two years after diagnosis [15]. Another study in 1462 Chinese patients with non-metastatic breast cancer reported a change in WCRF/AICR score from 3.2 at baseline to 3.9 at 18 months after diagnosis [14]. Since we observed changes in single lifestyle behaviours, a possible explanation for the unchanged WCRF/AICR score in our study could be that patients made simultaneous changes that resulted in higher adherence to one lifestyle behaviour together with worse adherence to another lifestyle behaviour. This may have resulted in no change in patients’ overall lifestyle score, which was also suggested in a previous study [15]. Another explanation may be that changes in singe lifestyle behaviours were too small to exceed the cut-off values of the components of the WCRF/AICR scoring system, since the magnitude of the changes was only small to moderate. It is unlikely that the stable WCRF/AICR score was explained by distinct subgroups of change (e.g., a group of patients with an increase and another group with a decrease in score), since no trajectories could be identified for the WCRF/AICR score. The largest change we found in single lifestyle behaviours was a decrease in red and processed meat intake. Other previous studies similarly reported a decrease in meat consumption over time in cancer survivors [15,39]. The second largest observed change was the decline in fruit and vegetable consumption. In contrast, most other studies in cancer survivors found an increase in fruit and vegetable intake [39]. Multiple reasons could explain this difference, including differences in study population, and method and timing of dietary assessment. Although we found a significant decrease in the proportion of current smokers after diagnosis, the majority of patients smoking at time of diagnosis continued smoking afterwards. Most other studies similarly concluded that a diagnosis with bladder cancer presents an opportunity for smoking cessation, but the reported smoking cessation rates were generally low (14–31%) [40,41,42].

This study was the first to identify trajectories of change for the WCRF/AICR score as well as for all single lifestyle behaviours. Few studies have previously applied a type of mixture modelling (e.g., group-based trajectory modelling, latent class trajectory modelling, longitudinal K-means) to longitudinal lifestyle data of recently diagnosed cancer patients. We found four observational studies that identified trajectories of physical activity [43,44,45,46] and one study that looked at separate trajectories of fruit and vegetables, dietary fat, and alcohol intake [20]. These studies focused on patients with either breast cancer or gynaecological cancer and used various types of mixture modelling approaches. Since different methods can lead to different trajectories being identified [47,48], comparing results across studies is difficult. Similar to our findings, the largest trajectory groups in other studies also showed no substantial change in lifestyle behaviours over time [20,40,41,42,43]. However, most studies also identified (smaller) trajectory groups that showed (both favourable and unfavourable) changes over time [20,43,44,45,46].

Our results showed that lifestyle behaviour hardly changed after diagnosis with NMIBC. There is a considerable gap between the lifestyle recommendations and the actual lifestyle behaviour of patients. Because a healthy lifestyle after cancer diagnosis might enhance long-term health outcomes, lifestyle support needs to be integrated into the care for (bladder) cancer patients. Previous findings of our cohort indicated that only a small proportion of patients (20%) received advice on physical activity, a healthy diet, or weight loss from their physician, while over 80% of the patients stated that they would find it beneficial, helpful, and encouraging to receive lifestyle advice from a physician [11]. Most smokers reported that they were advised to quit (70%), yet less than one out of three was offered help with smoking cessation [11]. Physicians should follow the EAU guidelines and provide all smokers with smoking cessation counselling. Since most smokers continued smoking after diagnosis, only advising to quit smoking seems insufficient, and physicians should also deliver smoking cessation interventions. Ultimately, interventions should also focus on training health care professionals to encourage cancer survivors to improve their lifestyle habits. Overall, future research is needed to find effective interventions to promote sustainable lifestyle changes and to explore the advantages of these changes for (bladder) cancer survivors. Correlates of (un)favourable lifestyle trajectories can help to identify patients who can benefit most from lifestyle support.

Some potential limitations of this study need to be addressed. Data were self-reported, which might be prone to measurement error. However, it is believed that the degree of under- and overreporting remains constant for every participant [49]. As a result, changes in lifestyle might be less influenced by this type of error. When we excluded observations with extreme energy and dietary intakes, results for lifestyle changes and trajectories were not considerably different. Furthermore, selection bias might be present. Patients who were interested in lifestyle may have been more likely to participate in this study. Since those who filled out at least two out of three questionnaires had a higher education level and a slightly higher baseline WCRF/AICR score compared to those who did not, caution is needed when generalizing these results to the total population of patients with NMIBC. Since missing data at one timepoint may have influenced our results, we performed sensitivity analyses by restricting to participants with full data at all three timepoints. This provided similar results, suggesting that the missing at random assumption of linear mixed models and latent class mixed models [50] was not violated. Lastly, lifestyle before diagnosis was assessed six weeks after diagnosis, which could have induced recall error.

This study has a number of strengths. First, a large number of patients with NMIBC was included in this study with data collected at multiple time points, providing a unique opportunity to study (changes in) lifestyle behaviours in this particular population. Furthermore, this was the first study to apply latent class trajectory modelling to such a variety of lifestyle behaviours as well as the overall WCRF/AICR score. An advantage of latent class trajectory modelling is that it allows for an unstructured variance–covariance structure for random effects that can differ across groups resulting in a better data fit, whereas the covariance structure in group-based trajectory modelling (Proc Traj in SAS) is limited to a common diagonal covariance structure [51]. Lastly, detailed information on covariates was available; thus, characteristics of trajectory groups could be explored.

## 5. Conclusions

Our study found that adherence to the 2018 WCRF/AICR lifestyle recommendations is generally low in patients with NMIBC and that their overall lifestyle remained constant in the first fifteen months after diagnosis. Changes were noted for most single lifestyle behaviours. No trajectories of change could be identified for the WCRF/AICR score, but trajectories were identified for all single lifestyle behaviours. Correlates differed per trajectory group, limiting direct applicability in designing lifestyle interventions. Due to the limited number of studies on lifestyle during the disease course, more knowledge is needed on how lifestyle (change) is related to clinical outcomes, such as recurrence, progression, and quality of life in patients with NMIBC. This knowledge is essential for formulating specific lifestyle recommendations for bladder cancer patients.

## Figures and Tables

**Table 1:** World Cancer Research Fund/American Institute for Cancer Research (WCRF/AICR) recommendations and adherence in patients with non-muscle invasive bladder cancer at baseline (*n* = 935) ^a^.

2018 WCRF/AICR Recommendations	Operationalization of Recommendations	Score	Adherence	
			*n*	%
1. Be a healthy weight	Body mass index (kg/m^2^) ^b^			
	18.5 to <25	1	318	34
	25 to <30	0.5	438	47
	<18.5 or ≥30	0	179	19
2. Be physically active	Moderate-to-vigorous physical activity ^c^ (min/week)			
	≥150	1	779	85
	75 to 150	0.5	57	6
	<75	0	81	9
3. Eat a diet rich in wholegrains, vegetables, fruit, and beans	Fruit and vegetables (g/day) ^d^			
	≥400	0.5	158	17
	200 to <400	0.25	413	44
	<200	0	360	39
	Total dietary fibre (g/day)			
	≥30	0.5	137	15
	15 to <30	0.25	669	72
	<15	0	125	13
4. Limit consumption of “fast foods” and other processed foods high in fat, starches or sugars	Percent of total kcal from ultra-processed foods (en%) ^e^			
	Tertile 1 < 26.6	1	310	33
	Tertile 2 26.6 to <36.2	0.5	311	33
	Tertile 3 ≥ 36.2	0	310	33
5. Limit consumption of red and processed meat	Red (g/week) and processed meat (g/week) ^f^			
	Red meat ≤ 500 and processed meat < 21	1	63	7
	Red meat ≤ 500 and processed meat 21 to <100	0.5	111	12
	Red meat > 500 or processed meat ≥ 100	0	757	81
6. Limit consumption of sugar sweetened drinks	Sugary drinks (g/day) ^g^			
	0	1	151	16
	>0 to ≤250	0.5	496	53
	>250	0	284	31
7. Limit alcohol consumption	Ethanol (g/day) ^h^			
	0	1	147	16
	>0 to ≤10	0.5	297	32
	>10	0	487	52

Abbreviations: en%, energy percentage; g, grams; kcal, kilocalorie; kg, kilograms; m, meters; min, minutes. ^a^ Following the standardized scoring system of the 2018 WCRF/AICR recommendations [31,32]. ^b^ Pre-diagnosis data were only available for BMI. Therefore, waist circumference was not used to score. ^c^ Moderate-to-vigorous physical activity includes leisure time (cycling, gardening, odd jobs, and sports with a metabolic equivalent value ≥ 3) and commuting (walking and cycling) activities. ^d^ Fruit and vegetables excluding potatoes and juices. ^e^ An adapted ultra-processed food (aUPF) variable was created based on the NOVA classification on food processing [33]. The definition of aUPF was altered to exclude food items already included in other components of the score and to be in line with the definitions from the WCRF/AICR 2018 Third Expert Report and national guidelines. Calculated as energy intake from aUPF versus total energy intake. Cut-offs were determined by cohort-specific tertiles at 6 weeks after diagnosis. Ultra-processed foods included ready-to-eat/heat foods (e.g., french fries, pizza, soups, meat substitutes, potato products, and fried fish), refined grains (e.g., white bread, pancakes, rice, pasta, and ready-to-eat breakfast cereals), snacks, sweets, and desserts (e.g., cakes, biscuits, confectionary, savoury snacks, custard or pudding, ice-cream, sugar added to coffee/tea, and diet drinks), spreads and sauces (e.g., peanut butter, chocolate spread, cream cheese, potato salad, mayonnaise, tomato ketchup) and some fats (hard margarine, solid cooking fats, frying fats). Not included were yoghurt, soft margarine, liquid cooking fats, brown/whole meal bread, sugary drinks, and processed meat. ^f^ Red meat included raw pork, beef, minced meat, fresh sausages, fresh hamburgers, liver, and game meat/other red meat. Processed meat included ready-to-eat/heat sausages, cold cuts/ham, and liver sausage/pate. ^g^ Sugary drinks included sugar-sweetened soft drinks, sweet dairy drinks, and fruit juices. Not included were diet drinks, tea/coffee with added sugar, and alcohol-free beer. Consumption of up to 250 g/month was defined as “zero” intake [32]. ^h^ Alcohol intake was based on alcoholic drinks (excluding alcohol-free beer). One drink corresponds to 10 g of ethanol. Consumption of up to 1 drink/month was defined as “zero” intake [32]. Part of the alcohol sub-score was based on national recommendations (limit to ≤10 g/d) [32].

**Table 2:** Baseline characteristics of 1076 Dutch patients with non-muscle invasive bladder cancer who were included or excluded in analyses of the WCRF/AICR score.

Characteristics ^a^	Overall *n* = 1076	Included ^b^ *n* = 935	Excluded ^b^ *n* = 141
Age at diagnosis, median (IQR), y	65.9 (61.0–72.0)	66.2 (61.0–72.0)	63.9 (58.0–72.0)
Male, *n* (%)	860 (80)	747 (80)	113 (80)
Educational level, *n* (%) ^c^			
Low	531 (49)	450 (48)	81 (57)
Medium	280 (26)	248 (27)	32 (23)
High	257 (24)	236 (25)	21 (15)
Work status, *n* (%)			
Employed	326 (30)	277 (30)	49 (35)
(Early) retirement	638 (59)	567 (61)	71 (50)
Unemployed	19 (2)	19 (2)	0 (0)
Occupationally disabled	49 (5)	42 (5)	7 (5)
Different	36 (3)	30 (3)	6 (4)
Living situation, *n* (%)			
With partner	763 (71)	675 (72)	88 (62)
With partner and kids	139 (13)	120 (13)	19 (14)
Alone with or without kids	153 (14)	128 (14)	25 (18)
Different	13 (1)	11 (1)	2 (1)
Body mass index, median (IQR), kg/m^2^	26.3 (24.3–29.0)	26.3 (24.3–29.0)	26.0 (24.8–29.1)
Body mass index, *n* (%)			
Underweight	10 (1)	8 (1)	2 (1)
Normal weight	353 (33)	319 (34)	34 (24)
Overweight	513 (48)	437 (47)	76 (54)
Obese	190 (18)	171 (18)	19 (14)
Smoking status before diagnosis, *n* (%)			
Never	188 (18)	168 (18)	20 (14)
Former	625 (58)	552 (59)	73 (52)
Current	256 (24)	215 (23)	41 (29)
WCRF/AICR score, median (IQR), 0–7	3.3 (2.8–3.8)	3.3 (2.8–3.8)	2.8 (2.5–3.5)
Presence of comorbidities, *n* (%)			
0	164 (15)	139 (15)	25 (18)
1	269 (25)	238 (25)	31 (22)
≥2	620 (58)	553 (59)	67 (48)
Stage, *n* (%)			
Ta	812 (76)	706 (76)	106 (75)
T1	236 (22)	204 (22)	32 (23)
Tis	28 (3)	25 (3)	3 (2)
Grade, *n* (%)			
1	242 (23)	207 (22)	35 (25)
2	525 (49)	454 (49)	71 (50)
3	306 (28)	271 (29)	35 (25)

Abbreviations: IQR, interquartile range; kg, kilograms; m, meters; WCRF/AICR, World Cancer Research Fund/American Institute for Cancer Research. ^a^ Where scores do not total 100%, this is because of missing values. ^b^ Participants who were included completed ≥2 assessments of the WCRF/AICR score at six weeks (T6wk), three months (T3mo), and/or fifteen months (T15mo) after diagnosis. Participants who were excluded completed 0 or 1 assessments of the WCRF/AICR score (43 (31%) completed 0 assessments, 89 (63%) completed only T6wk, 7 (5%) completed only T3mo, and 2 (1%) completed only T15mo). ^c^ Low: primary, secondary, and vocational education; medium: intermediate vocational education and higher general secondary education; high: higher vocational.

**Table 3:** Change in lifestyle behaviours in the first fifteen months after diagnosis with non-muscle-invasive bladder cancer.

Lifestyle Behaviour	*n*	Baseline ^a^	3 Months after Diagnosis ^a^	15 Months after Diagnosis ^a^	Change ^b^	Effect Size ^b,c^	*p* Trend ^d^
WCRF/AICR score	935	3.3 (3.2, 3.3)	3.2 (3.1, 3.3)	3.3 (3.2, 3.3)	0.0 (−0.1, 0.0)	−0.04 (−0.15, 0.06)	0.96
BMI (kg/m^2^)	960	26.8 (26.6, 27.1)	26.9 (26.7, 27.2)	26.9 (26.6, 27.2)	0.1 (0.0, 0.2)	0.14 (−0.07, 0.35)	0.31
Physical activity (min/week)	961	750 (708, 792)	637 (595, 679)	665 (620, 709)	−85 (−136, −35)	−0.19 (−0.29, −0.10)	0.01
Fruit and vegetables (g/day)	949	261 (253, 271)	223 (214, 231)	224 (215, 234)	−38 (−48, −27)	−0.46 (−0.57, −0.35)	<0.001
Dietary fibre (g/day)	949	22.6 (22.2, 23.1)	21.7 (21.2, 22.2)	21.3 (20.8, 21.7)	−1.4 (−1.9, −0.9)	−0.34 (−0.45, −0.23)	<0.001
Ultra-processed foods (en%) ^e^	949	31.8 (31.2, 32.5)	34.5 (33.8, 35.2)	34.0 (33.3, 34.8)	2.2 (1.4, 2.9)	0.38 (0.28, 0.50)	<0.001
Red and processed meat (g/week)	949	704 (681, 727)	634 (611, 656)	608 (586, 630)	−96 (−121, −71)	−0.49 (−0.60, −0.38)	<0.001
Sugary drinks (g/day)	949	215 (197, 234)	202 (184, 221)	187 (169, 205)	−28 (−50, −7)	−0.15 (−0.25, −0.05)	<0.01
Alcohol (g/day)	949	16.1 (14.9, 17.3)	14.3 (13.1, 15.4)	13.9 (12.8, 14.9)	−2.3 (−3.2, −1.3)	−0.27 (−0.37, −0.18)	<0.001
Smoking (%)	966	23.2 (20.5, 25.8)	16.3 (14.0, 18.7)	15.0 (12.6, 17.5)	−8.1 (−12, −4.5)	−0.44 (−0.65, −0.24)	<0.001

Abbreviations: BMI, body mass index; g, grams; kg, kilograms; m, meters; min, minutes; WCRF/AICR, World Cancer Research Fund/American Institute for Cancer Research. ^a^ Estimated marginal means (95% CI). ^b^ Fifteen months after diagnosis—baseline. ^c^ Effect size (*d*) of 0.20 is considered a small effect size, 0.50 is a moderate effect size, 0.80 is a large effect size. ^d^ *p* for trend values were based on the linear mixed models with three repeated measures and time (continuous). For smoking, we used a logistic mixed model. ^e^ An adapted ultra-processed food (aUPF) variable was created based on the NOVA classification on food processing [33]. The definition of aUPF was altered to exclude food items already included in other components of the score and to be in line with the definitions from the WCRF/AICR 2018 Third Expert Report and national guidelines. Calculated as energy intake from aUPF versus total energy intake. Cut-offs were determined by cohort-specific tertiles at 6 weeks after diagnosis. Ultra-processed foods included ready-to-eat/heat foods (e.g., french fries, pizza, soups, meat substitutes, potato products, and fried fish), refined grains (e.g., white bread, pancakes, rice, pasta, and ready-to-eat breakfast cereals), snacks, sweets, and desserts (e.g., cakes, biscuits, confectionary, savoury snacks, custard or pudding, ice-cream, sugar added to coffee/tea, and diet drinks), spreads and sauces (e.g., peanut butter, chocolate spread, cream cheese, potato salad, mayonnaise, tomato ketchup) and some fats (hard margarine, solid cooking fats, frying fats). Not included were yoghurt, soft margarine, liquid cooking fats, brown/whole meal bread, sugary drinks, and processed meat.

## Data Availability

The data presented in this study are available on request from the corresponding author.

## Supplementary Materials

The following supporting information can be downloaded at: https://www.mdpi.com/article/10.3390/cancers14040960/s1, Table S1: Model-specific adequacy assessments of latent trajectory class models, Table S2: Group-specific adequacy assessments of latent trajectory class, Table S3: Multivariable multinomial logistic regression of correlates of body mass index trajectories (*n* = 960), Table S4: Multivariable multinomial logistic regression of correlates of moderate-to-vigorous physical activity trajectories (*n* = 961), Table S5: Multivariable multinomial logistic regression of correlates of fruit and vegetables intake trajectories (*n* = 949), Table S6: Multivariable multinomial logistic regression of correlates of dietary intake trajectories (*n* = 949), Table S7: Multivariable multinomial logistic regression of correlates of ultra-processed food intake trajectories (*n* = 949), Table S8: Multivariable multinomial logistic regression of correlates of red and processed meat intake trajectories (*n* = 949), Table S9: Multivariable multinomial logistic regression of correlates of sugary drink intake trajectories (*n* = 949), Table S10: Multivariable multinomial logistic regression of correlates of alcohol intake trajectories (*n* = 949), Table S11: Changes in lifestyle behaviours in the first fifteen months after diagnosis in patients with full data at all three time points, Table S12: Changes in lifestyle behaviours in the first fifteen months after diagnosis after excluding observations where extreme energy or dietary intakes were reported.
