# Supplementary material for: Limited Changes in Lifestyle Behaviours after Non-Muscle Invasive Bladder Cancer Diagnosis
Source: Cancers (Basel). 2022 Feb 15;14(4):960. doi: 10.3390/cancers14040960 (PMC8869990; doi:10.3390/cancers14040960)
Supplement: Supplementary file 1 [file cancers-14-00960-s001.zip › cancers-1530453-supplementary.pdf]

cancers

MDPI

Supplementary materials

Limited Changes in Lifestyle Behaviours after Non-Muscle Invasive Bladder Cancer Diagnosis

Ivy Beeren, Liesbeth de Goeij, Rana Dandis, Nikoletta Vidra, Moniek van Zutphen, J. Alfred Witjes, Ellen Kampman, Lambertus A.L.M. Kiemeney, and Alina Vrieling

Table S1. Model-specific adequacy assessments of latent trajectory class models

Number of groups[a]	BIC	AIC	Entropy	Proportions per group (%)						
				1	2	3	4	5	6	7
**WCRF/AICR score (0-7), n=935**										
1 (selected)	5896	5847	1.00	100						
2	5898	5825	0.26	29	71					
3	5919	5822	0.64	7	5	88				
4	5950	5829	0.39	16	31	21	32			
5	5974	5829	0.54	36	2	17	9	35		
6	5987	5818	0.56	23	7	23	11	3	33	
7	6037	5843	0.47	9	10	12	13	7	43	6
**Body mass index (kg/m^2), n=960**										
1	9684	9635	1.00	100						
2 (selected)	9126	9053	0.61	79	21					
3	9098	9001	0.55	41	56	3				
4	9038	8916	0.66	2	29	63	6			
5	9030	8884	0.69	2	27	4	61	6		
6	9090	8920	0.70	1	0	10	74	4	11	
7	9106	8911	0.67	2	29	0	60	1	2	6
**Moderate-to-vigorous physical activity (minutes/week), n=960**										
1	39520	39461	1.00	100						
2	39181	39098	0.78	82	18					

3	39170	39073	0.60	45	35	20				
4 (selected)	39166	39035	0.54	17	33	35	15			
5	39175	39019	0.58	16	23	24	23	14		
6	39187	39007	0.61	14	8	25	23	16	14	
7	39297	39093	0.55	10	15	18	13	16	11	17
**Dietary fibre intake (g/day), n=949**										
1	17343	17294	1.00	100						
2 (selected)	16982	16909	0.67	89	11					
3	16955	16858	0.54	2	59	39				
4	16966	16844	0.56	19	67	13	1			
5	16988	16842	0.67	36	4	54	4	2		
6	17049	16893	0.64	1	13	28	51	5	2	
7	17024	16829	0.55	1	16	22	5	10	8	38
**Fruit and vegetables intake (g/day), n=949**										
1	33373	33324	1.00	100						
2	32989	32916	0.54	65	35					
3 (selected)	32855	32758	0.65	5	59	36				
4	32876	32755	0.72	4	55	1	40			
5	32834	32689	0.72	2	48	34	1	15		
6	32836	32666	0.70	0	6	42	15	1	36	
7	32897	32703	0.76	1	2	47	34	0	1	15
**Ultra-processed food intake (en%), n=949**										
1	19281	19232	1.00	100						
2 (selected)	19243	19170	0.47	91	9					
3	19217	19120	0.49	1	61	39				
4	19223	19101	0.51	48	36	15	0			
5	19255	19109	0.56	53	29	16	2	0		
6	19278	19108	0.59	10	0	46	13	4	27	
7	19316	19121	0.63	39	0	24	22	4	2	8
**Red and processed meat intake (g/week), n=949**										
1	38132	38084	1.00	100						
2	38166	38094	1.00	0	100					

3 (selected)	37766	37668	0.73	81	14	5				
4	37906	37785	0.78	1	14	0	85			
5	37982	37837	0.84	8	0	0	90	2		
6	37744	37574	0.70	5	7	1	1	53	33	
7	37851	37657	0.74	1	2	5	48	0	1	43
**Sugary drinks intake (g/day), n=949**										
1	31588	31530	1.00	100						
2	31133	31051	0.72	55	45					
3 (selected)	31029	30923	0.79	58	6	36				
4	30984	30853	0.71	6	36	25	33			
5	30820	30665	0.81	6	16	33	24	21		
6	30750	30571	0.79	6	13	30	16	24	11	
7	31059	30855	0.67	14	14	19	17	6	10	20
**Alcohol intake (g/day), n=949**										
1	17455	17397	1.00	100						
2	16835	16752	0.69	53	47					
3	16809	16703	0.71	38	20	42				
4 (selected)	15124	14993	0.75	16	54	18	12			
5	15207	15052	0.74	28	33	14	13	12		
6	16116	15936	0.79	5	59	11	2	15	8	
7	15338	15135	0.79	13	35	6	24	9	1	12

Abbreviations: AIC, Akaike information criterion; BIC, Bayesian information criterion; g, grams; kg, kilograms; m, meter; WCRF/AICR, World Cancer Research Fund/American Institute for Cancer Research.

[a] Models with the lowest BIC value and with all groups including ≥5% of the total study population were favoured. When those criteria were both met and another model was selected, this is due to overlapping curves and/or confidence intervals of trajectory groups.

Table S2. Group-specific adequacy assessments of latent trajectory class models

Group	Proportion (%)	AvePP[a]	OCC[b]
**WCRF/AICR score (0-7), n=935**			
Medium maintainers	100	n.a.	n.a.
**Body mass index (kg/m²), n=960**			
High maintainers	79	0.90	3.2
Medium maintainers	21	0.86	16.1
**Moderate-to-vigorous physical activity (minutes/week), n=961**			
High increasers	17	0.70	6.9
High decreasers	33	0.69	5.9
Medium maintainers	35	0.72	5.8
Medium decreasers	15	0.94	77.5
**Fruit and vegetables intake (g/day), n=949**			
High decreasers	5	0.82	60.9
Medium decreasers	59	0.87	4.8
Low maintainers	36	0.80	7.9
**Dietary fibre intake (g/day), n=949**			
High decreasers	11	0.87	34.6
Medium maintainers	89	0.93	2.5
**Ultra-processed food intake (en%), n=949**			
High increasers	9	0.75	13.1
Medium increasers	91	0.89	1.5
**Red and processed meat intake (g/week), n=949**			
High decreasers	14	0.84	20.6
Medium maintainers	81	0.90	2.8
Low maintainers	5	0.87	133.1
**Sugary drinks intake (g/day), n=949**			
High decreasers	58	0.90	7.4
Medium decreasers	6	0.89	110.6
Low increasers	36	0.93	20.9
**Alcohol intake (g/day), n=949**			
High decreasers	54	0.90	8.4

Medium decreasers	18	0.82	15.9
Low maintainers	16	0.67	11.0
Zero maintainers	12	1.00	25638.3

Abbreviations: AvePP, average posterior probability; OCC, odds of correct classification; WCRF/AICR, World Cancer Research Fund/American Institute for Cancer Research.

[a] The closer the AvePP are to 1, the better the model fit. An AvePP greater than 0.7 for all groups is generally recommended.

[b] The higher the OCC value, the better the model fit. An OCC value greater than 5.0 for all groups is generally recommended.

Table S3. Multivariable multinomial logistic regression of correlates of body mass index trajectories (n=960)

Correlates	High maintainers n=200	Medium maintainers n=760	
		**OR**	**95% CI**
Age (ref = 65+)	Ref	0.85	0.59, 1.24
Gender (ref = male)	Ref	0.89	0.60, 1.32
Education (ref = low)[b]			
Medium	Ref	0.96	0.66, 1.41
High	Ref	1.24	0.82, 1.88
Living situation (ref = with partner)			
With partner and kids	Ref	0.84	0.50, 1.41
Alone with or without kids	Ref	0.76	0.48, 1.18
Smoking (ref = never)			
Current	Ref	0.72	0.43, 1.20
Former	Ref	0.85	0.54, 1.35
Tumour stage (ref = Ta)			
Tis or T1	Ref	0.81	0.56, 1.16
Comorbidities (ref = 0)			
1	Ref	0.77	0.44, 1.34
≥2	Ref	0.64	0.39, 1.05

Abbreviations: CI, confidence interval; ref, reference; OR, odds ratio.

[a] Bold indicates significant results ($p < 0.05$).

[b] Low: primary, secondary, and vocational education, medium: intermediate vocational education and higher general secondary education, high: higher vocational education and university.

Table S4. Multivariable multinomial logistic regression of correlates of moderate-to-vigorous physical activity (n=961)

	Medium decreasers n=145	High increasers n=159[a]		High decreasers n=317[a]		Medium maintainers n=340[a]	
**Correlates**		**OR**	**95% CI**	**OR**	**95% CI**	**OR**	**95% CI**
Age (ref = 65+)	Ref	1.18	0.66, 2.11	1.31	0.79, 2.20	**1.81**	**1.11, 2.96**
Gender (ref = male)	Ref	**0.27**	**0.15, 0.51**	**0.33**	**0.20, 0.55**	0.70	0.44, 1.11
Education (ref = low)[b]							
Medium	Ref	1.36	0.75, 2.48	**1.81**	**1.06, 3.11**	**1.75**	**1.03, 2.96**
High	Ref	1.12	0.59, 2.10	**1.84**	**1.06, 3.18**	1.67	0.97, 2.87
Living situation (ref = with partner)							
With partner and kids	Ref	0.59	0.23, 1.53	0.87	0.39, 1.92	1.42	0.68, 2.98
Alone with or without kids	Ref	**0.45**	**0.23, 0.88**	**0.36**	**0.20, 0.64**	0.70	0.42, 1.18
Body mass index (ref = normal weight)[c,d]							
Overweight	Ref	1.02	0.59, 1.78	1.00	0.61, 1.62	1.04	0.64, 1.69
Obese	Ref	0.68	0.35, 1.34	**0.45**	**0.25, 0.83**	0.80	0.45, 1.42
Smoking (ref = never)							
Current	Ref	0.56	0.26, 1.25	**0.49**	**0.24, 0.99**	**0.44**	**0.23, 0.85**
Former	Ref	1.00	0.49, 2.04	1.16	0.62, 2.18	0.60	0.33, 1.09
Tumour stage (ref = Ta)							
Tis or T1	Ref	**0.52**	**0.30, 0.89**	**0.56**	**0.35, 0.89**	**0.61**	**0.39, 0.97**
Comorbidities (ref = 0)							
1	Ref	1.11	0.50, 2.50	1.42	0.68, 2.93	1.32	0.65, 2.66
≥2	Ref	0.82	0.40, 1.66	0.98	0.52, 1.86	0.82	0.45, 1.51

Abbreviations: CI, confidence interval; ref, reference; OR, odds ratio.

[a] Bold indicates significant results ($p < 0.05$).

[b] Low: primary, secondary, and vocational education, medium: intermediate vocational education and higher general secondary education, high: higher vocational education and university.

[c] Normal weight: body mass index <24.9 kg/m^2, overweight: body mass index 25.0–29.9 kg/m^2, obese: body mass index ≥30.0 kg/m^2.

[d] n=8 participants were included with body mass index <18.5 kg/m^2.

Table S5. Multivariable multinomial logistic regression of correlates of fruit and vegetables intake trajectories (n=949)

	**Low maintainers**	**High decreasers**		**Medium decreasers**	
	**n=345**	**n=50[a]**		**n=554[a]**	
**Correlates**		**OR**	**95% CI**	**OR**	**95% CI**
Age (ref = 65+)	Ref	0.49	0.22, 1.08	0.90	0.64, 1.26
Gender (ref = male)	Ref	2.13	1.00, 4.55	1.55	1.07, 2.26
Education (ref = low)[b]	Ref				
Medium	Ref	**2.34**	**1.08, 5.06**	1.12	0.80, 1.59
High	Ref	**2.79**	**1.31, 5.93**	1.03	0.72, 1.48
Living situation (ref = with partner)	Ref				
With partner and kids	Ref	0.14	0.02, 1.11	0.67	0.42, 1.05
Alone with or without kids	Ref	**2.19**	**1.03, 4.66**	1.26	0.82, 1.94
Body mass index (ref = normal weight)[c,d]	Ref				
Overweight	Ref	0.61	0.30, 1.25	0.83	0.60, 1.14
Obese	Ref	1.35	0.60, 3.07	0.78	0.51, 1.19
Smoking (ref = never)	Ref				
Current	Ref	0.59	0.23, 1.52	**0.47**	**0.30, 0.73**
Former	Ref	1.04	0.46, 2.36	1.18	0.80, 1.74
Tumour stage (ref = Ta)	Ref				
Tis or T1	Ref	1.08	0.52, 2.21	1.17	0.84, 1.62
Comorbidities (ref = 0)	Ref				
1	Ref	0.87	0.32, 2.37	0.63	0.39, 1.01
≥2	Ref	0.53	0.21, 1.37	**0.63**	**0.41, 0.98**

Abbreviations: CI, confidence interval; ref, reference; OR, odds ratio.

[a] Bold indicates significant results ($p < 0.05$).

[b] Low: primary, secondary, and vocational education, medium: intermediate vocational education and higher general secondary education, high: higher vocational education and university.

[c] Normal weight: body mass index <24.9 kg/m^2, overweight: body mass index 25.0–29.9 kg/m^2, obese: body mass index ≥30.0 kg/m^2.

[d] n=8 participants were included with body mass index <18.5 kg/m^2.

Table S6. Multivariable multinomial logistic regression of correlates of dietary fibre intake trajectories (n=949)

Correlates	Medium maintainers n=518	High decreasers n=152[a]	
		OR	95% CI
Age (ref = 65+)	Ref	1.16	0.70, 1.92
Gender (ref = male)	Ref	**0.40**	**0.21, 0.78**
Education (ref = low)[b]			
Medium	Ref	1.44	0.87, 2.38
High	Ref	1.12	0.66, 1.90
Living situation (ref = with partner)			
With partner and kids	Ref	0.93	0.46, 1.89
Alone with or without kids	Ref	1.43	0.80, 2.57
Body mass index (ref = normal weight)[c,d]			
Overweight	Ref	0.51	0.32, 0.81
Obese	Ref	0.85	0.47, 1.54
Smoking (ref = never)			
Current	Ref	0.57	0.30, 1.09
Former	Ref	0.64	0.38, 1.09
Tumour stage (ref = Ta)			
Tis or T1	Ref	0.89	0.54, 1.47
Comorbidities (ref = 0)			
1	Ref	1.26	0.64, 2.50
≥2	Ref	1.87	0.92, 3.78

Abbreviations: CI, confidence interval; ref, reference; OR, odds ratio.

[a] Bold indicates significant results ($p < 0.05$).

[b] Low: primary, secondary, and vocational education, medium: intermediate vocational education and higher general secondary education, high: higher vocational education and university.

[c] Normal weight: body mass index <24.9 kg/m^2, overweight: body mass index 25.0–29.9 kg/m^2, obese: body mass index ≥30.0 kg/m^2.

[d] n=8 participants were included with body mass index <18.5 kg/m^2.

Table S7. Multivariable multinomial logistic regression of correlates of ultra-processed food intake trajectories (n=949)

	**High increasers**	**Medium increasers**	
	**n=83**	**n=866[a]**	
**Correlates**		**OR**	**95% CI**
Age (ref = 65+)	Ref	0.88	0.52, 1.51
Gender (ref = male)	Ref	1.01	0.57, 1.80
Education (ref = low)[b]	Ref		
Medium	Ref	1.05	0.59, 1.87
High	Ref	0.72	0.41, 1.25
Living situation (ref = with partner)	Ref		
With partner and kids	Ref	0.75	0.36, 1.56
Alone with or without kids	Ref	0.58	0.32, 1.05
Body mass index (ref = normal weight)[c,d]	Ref		
Overweight	Ref	1.33	0.80, 2.22
Obese	Ref	1.30	0.66, 2.56
Smoking (ref = never)	Ref		
Current	Ref	**0.41**	**0.19, 0.85**
Former	Ref	0.80	0.40, 1.62
Tumour stage (ref = Ta)	Ref		
Tis or T1	Ref	0.91	0.54, 1.55
Comorbidities (ref = 0)	Ref		
1	Ref	0.76	0.34, 1.68
≥2	Ref	0.67	0.32, 1.39

Abbreviations: CI, confidence interval; ref, reference; OR, odds ratio.

[a] Bold indicates significant results ($p < 0.05$).

[b] Low: primary, secondary, and vocational education, medium: intermediate vocational education and higher general secondary education, high: higher vocational education and university.

[c] Normal weight: body mass index <24.9 kg/m^2, overweight: body mass index 25.0–29.9 kg/m^2, obese: body mass index ≥30.0 kg/m^2.

[d] n=8 participants were included with body mass index <18.5 kg/m^2.

Table S8. Multivariable multinomial logistic regression of correlates of red and processed meat intake trajectories (n=949)

	High decreasers n=134	Medium maintainers n=776[a]		Low maintainers n=49[a]	
**Correlates**		**OR**	**95% CI**	**OR**	**95% CI**
Age (ref = 65+)	Ref	1.03	0.65, 1.64	0.65	0.27, 1.58
Gender (ref = male)	Ref	**3.14**	**1.66, 5.93**	**7.32**	**2.97, 18.08**
Education (ref = low)[b]					
Medium	Ref	1.45	0.93, 2.27	0.85	0.30, 2.39
High	Ref	**3.43**	**1.87, 6.30**	**6.70**	**2.71, 16.58**
Living situation (ref = with partner)					
With partner and kids	Ref	0.93	0.48, 1.77	1.43	0.42, 4.86
Alone with or without kids	Ref	1.26	0.68, 2.35	**3.02**	**1.20, 7.59**
Body mass index (ref = normal weight)[c,d]					
Overweight	Ref	0.82	0.52, 1.29	**0.29**	**0.12, 0.66**
Obese	Ref	0.59	0.34, 1.02	**0.31**	**0.11, 0.88**
Smoking (ref = never)					
Current	Ref	0.42	0.22, 0.82	**0.13**	**0.04, 0.42**
Former	Ref	0.77	0.41, 1.44	0.46	0.18, 1.14
Tumour stage (ref = Ta)					
Tis or T1	Ref	0.84	0.54, 1.30	0.59	0.25, 1.41
Comorbidities (ref = 0)					
1	Ref	1.09	0.58, 2.06	0.79	0.25, 2.50
≥2	Ref	1.07	0.60, 1.90	1.04	0.38, 2.88

Abbreviations: CI, confidence interval; ref, reference; OR, odds ratio.

[a] Bold indicates significant results ($p < 0.05$).

[b] Low: primary, secondary, and vocational education, medium: intermediate vocational education and higher general secondary education, high: higher vocational education and university.

[c] Normal weight: body mass index <24.9 kg/m^2, overweight: body mass index 25.0–29.9 kg/m^2, obese: body mass index ≥30.0 kg/m^2.

[d] n=8 participants were included with body mass index <18.5 kg/m^2.

Table S9. Multivariable multinomial logistic regression of correlates of sugary drink intake trajectories (n=949)

	High decreasers	Medium decreasers		Low increasers	
	n=548	n=59[a]		n=342[a]	
**Correlates**		**OR**	**95% CI**	**OR**	**95% CI**
Age (ref = 65+)	Ref	**1.90**	**1.04, 3.47**	**0.64**	**0.45, 0.90**
Gender (ref = male)	Ref	1.06	0.51, 2.18	**1.87**	**1.32, 2.65**
Education (ref = low)[b]	Ref				
Medium	Ref	0.99	0.52, 1.88	**1.45**	**1.03, 2.04**
High	Ref	0.47	0.21, 1.04	1.03	0.72, 1.47
Living situation (ref = with partner)	Ref				
With partner and kids	Ref	0.68	0.29, 1.62	0.96	0.59, 1.55
Alone with or without kids	Ref	1.08	0.50, 2.37	0.81	0.54, 1.24
Body mass index (ref = normal weight)[c,d]	Ref				
Overweight	Ref	1.37	0.70, 2.67	0.81	0.59, 1.11
Obese	Ref	1.84	0.82, 4.14	0.87	0.58, 1.32
Smoking (ref = never)	Ref				
Current	Ref	0.89	0.38, 2.11	1.15	0.73, 1.82
Former	Ref	0.95	0.45, 2.00	1.32	0.89, 1.95
Tumour stage (ref = Ta)	Ref				
Tis or T1	Ref	**1.89**	**1.06, 3.36**	0.89	0.64, 1.25
Comorbidities (ref = 0)	Ref				
1	Ref	0.68	0.29, 1.61	1.18	0.74, 1.88
≥2	Ref	0.77	0.37, 1.62	1.08	0.71, 1.65

Abbreviations: CI, confidence interval; ref, reference; OR, odds ratio.

[a] Bold indicates significant results ($p < 0.05$).

[b] Low: primary, secondary, and vocational education, medium: intermediate vocational education and higher general secondary education, high: higher vocational education and university.

[c] Normal weight: body mass index <24.9 kg/m^2, overweight: body mass index 25.0–29.9 kg/m^2, obese: body mass index ≥30.0 kg/m^2.

[d] n=8 participants were included with body mass index <18.5 kg/m^2.

Table S10. Multivariable multinomial logistic regression of correlates of alcohol intake trajectories (n=949)

	High decreasers	Low maintainers		Medium decreasers		Stable non-consumers	
	n=518	n=152[a]		n=168[a]		n=111[a]	
Correlates		OR	95% CI	OR	95% CI	OR	95% CI
Age (ref = 65+)	Ref	1.06	0.68, 1.66	0.89	0.58, 1.37	0.96	0.57, 1.61
Gender (ref = male)	Ref	**2.56**	**1.65, 3.98**	0.90	0.54, 1.52	**3.09**	**1.90, 5.02**
Education (ref = low)[b]							
Medium	Ref	0.95	0.60, 1.49	0.91	0.59, 1.41	0.86	0.51, 1.46
High	Ref	0.61	0.37, 1.01	0.77	0.49, 1.19	**0.53**	**0.29, 0.96**
Living situation (ref = with partner)							
With partner and kids	Ref	1.37	0.76, 2.46	0.74	0.39, 1.42	1.29	0.62, 2.66
Alone with or without kids	Ref	1.09	0.62, 1.91	0.81	0.47, 1.42	1.76	1.00, 3.08
Body mass index (ref = normal weight)[c,d]							
Overweight	Ref	1.21	0.78, 1.88	1.00	0.68, 1.49	0.98	0.59, 1.63
Obese	Ref	**1.93**	**1.12, 3.32**	1.03	0.59, 1.79	1.64	0.89, 3.02
Smoking (ref = never)							
Current	Ref	0.62	0.34, 1.11	1.19	0.67, 2.10	1.15	0.60, 2.21
Former	Ref	**0.57**	**0.35, 0.92**	0.80	0.49, 1.31	0.58	0.32, 1.05
Tumour stage (ref = Ta)							
Tis or T1	Ref	0.83	0.53, 1.33	**1.49**	**1.01, 2.20**	1.09	0.65, 1.83
Comorbidities (ref = 0)							
1	Ref	0.74	0.40, 1.35	0.87	0.49, 1.54	1.57	0.70, 3.50
≥2	Ref	0.86	0.51, 1.47	0.99	0.59, 1.67	1.77	0.84, 3.72

Abbreviations: CI, confidence interval; ref, reference; OR, odds ratio.

[a] Bold indicates significant results ($p < 0.05$).

[b] Low: primary, secondary, and vocational education, medium: intermediate vocational education and higher general secondary education, high: higher vocational education and university.

[c] Normal weight: body mass index <24.9 kg/m^2, overweight: body mass index 25.0–29.9 kg/m^2, obese: body mass index ≥30.0 kg/m^2.

[d] n=8 participants were included with body mass index <18.5 kg/m^2.

Table S11. Changes in lifestyle behaviours in the first fifteen months after diagnosis in patients with full data at all three time points

Lifestyle behaviours	n	Baseline[a]	3 months after diagnosis[a]	15 months after diagnosis[a]	Change[b]	Effect size[b,c]	P trend[d]
WCRF/AICR score	737	3.3 (3,2 3.4)	3.2 (3.1, 3.3)	3.3 (3.2, 3.3)	0.0 (-0.1, 0.0)	-0.06 (-0.18, -0.05)	0.90
Body mass index (kg/m^2)	814	26.9 (26.6, 27.2)	27.0 (26.7, 27.3)	26.9 (26.6, 27.2)	0.0 (-0.1, 0.1)	0.09 (-0.24, 0.12)	0.68
Physical activity (min/week)	784	742 (695, 789)	627 (580, 673)	647 (603, 690)	-96 (-148, -43)	-0.23 (-0.33, -0.12)	<0.01
Fruit & vegetables (g/day)	795	267 (257, 277)	226 (216, 235)	228 (218, 237)	-39 (-51, -28)	-0.49 (-0.61, -0.37)	<0.001
Dietary fibre (g/day)	795	22.8 (22.3, 23.3)	21.7 (21.2, 22.3)	21.4 (20.9, 21.9)	-1.4 (-2.0, -0.9)	-0.35 (-0.46, -0.24)	<0.001
Ultra-processed foods (en%)	795	32 (31, 32)	34 (34, 35)	34 (33, 35)	2.3 (1.5, 3.1)	0.41 (0.30, -0.53)	<0.001
Red & processed meat (g/week)	795	695 (671, 719)	629 (606, 653)	603 (580, 626)	-92 (-118, -66)	-0.48 (-0.59, -0.36)	<0.001
Sugary drinks (g/day)	795	214 (194, 235)	204 (184, 224)	184 (166, 203)	-30 (-53, -7.3)	-0.16 (0.26, 0.06)	<0.01
Alcohol (g/day)	795	16.5 (15.2, 17.8)	14.4 (13.2, 15.7)	14.1 (12.9, 15.3)	-2.4 (-3.4, -1.4)	-0.29 (-0.38, -0.18)	<0.001
Current smokers (%)	817	22.0 (19.2, 24.9)	14.7 (12.3, 17.1)	14.9 (12.5, 17.4)	-7.1 (-11.9, -4.2)	-0.41 (-0.63, -0.19)	<0.01

Abbreviations: g, grams; kg, kilograms; m, meters; min, minutes; WCRF/AICR World Cancer Research Fund/American Institute of Cancer Research.

[a] Estimated marginal means (95% CI).

[b] Fifteen months after diagnosis – baseline.

[c] Effect size (*d*) of 0.20 is considered a small effect size, 0.50 is a moderate effect size, 0.80 is a large effect size.

[d] P for trend values were based on the linear mixed models with three repeated measures and time (continuous). For smoking, we used a logistic mixed model.

Table S12. Changes in lifestyle behaviours in the first fifteen months after diagnosis after excluding observations where extreme energy or dietary intakes[a] were reported

Lifestyle behaviours	n	Baseline[b]	3 months after diagnosis[b]	15 months after diagnosis[b]	Change[c]	Effect size[c,d]	P trend[e]
WCRF/AICR score	867	3.3 (3.2, 3.3)	3.2 (3.1, 3.3)	3.3 (3.2, 3.3)	0.0 (-0.1, 0.0)	-0.04 (-0.16, 0.07)	0.85
Fruit & vegetables (g/d)	891	254 (246, 263)	219 (211, 228)	219 (210, 227)	-36 (-45, -26)	-0.49 (-0.59, -0.38)	<0.001
Dietary fibre (g/d)	891	22.0 (21.6, 22.5)	21.2 (20.8, 21.7)	20.9 (20.4, 21.3)	-1.2 (-1.6, -0.7)	-0.34 (-0.46, -0.23)	<0.001
Ultra-processed foods (en%)	891	31.7 (31.1, 32.4)	34.5 (33.8, 35.1)	34.0 (33.3, 34.7)	2.2 (1.5, 3.0)	0.40 (0.29, 0.51)	<0.001
Red & processed meat (g/wk)	891	667 (657, 698)	616 (596, 636)	589 (568, 609)	-89 (-111, -66)	-0.49 (-0.60, -0.39)	<0.001
Sugary drinks (g/d)	891	188 (174, 202)	172 (158, 186)	159 (146, 173)	-29 (-45, -13)	-0.22 (-0.32, -0.11)	<0.001
Alcohol (g/d)	891	14.3 (13.4, 15.3)	13.2 (12.3, 14.2)	12.5 (11.6, 13.4)	-1.8 (-2.5, -1.1)	-0.34 (-0.45, -0.23)	<0.001

Abbreviations: g, grams; kg, kilograms; m, meters; min, minutes; WCRF/AICR World Cancer Research Fund/American Institute of Cancer Research.

[a] For energy intake: <500 or >3500 kcal/day for women, <800 or >4200 kcal/day for men and for dietary intake: ≥3 standard deviations from the mean.

[b] Estimated marginal means (95% CI).

[c] Fifteen months after diagnosis – baseline.

[d] Effect size (*d*) of 0.20 is considered a small effect size, 0.50 is a moderate effect size, 0.80 is a large effect size.

[e] P for trend values were based on the linear mixed models with three repeated measures and time (continuous).
